# The biology, function, and applications of exosomes as novel biomarkers for *in-vivo* drug exposure variability

**DOI:** 10.3389/fphar.2026.1822691

**Published:** 2026-05-18

**Authors:** Yihui Yang, Zhujun Yu, Wei Dou, Shuping Shi, Bo Zhang, Xin Liu

**Affiliations:** 1 Department of Pharmacy, Peking Union Medical College Hospital, Chinese Academy of Medical Sciences and Peking Union Medical College, Beijing, China; 2 State Key Laboratory of Complex Severe and Rare Diseases, Peking Union Medical College Hospital, Beijing, China; 3 Department of Clinical Pharmacy, Shenyang Pharmaceutical University, Shenyang, China

**Keywords:** biomarkers, drug-metabolizing enzymes, exosomes, in vivo drug exposure variability, therapeutic drug monitoring

## Abstract

Exosomes have become one of the hotspots in life science research in recent years. As an important carrier of bio-information substances, they can mediate intercellular communication by carrying various bioactive substances, thereby activating specific signal transduction pathways within cells and ultimately regulating cell phenotypes and functions. Among the abundant cargoes carried by exosomes, drug-metabolizing enzymes have gradually attracted increasing attention from researchers. More and more evidence suggests that the abundance and activity levels of these specific drug-metabolizing enzymes encapsulated in exosomes can often reflect the dynamic gene expression in their parent cells, which is of great significance for studying inter-individual variability in drug exposure. Based on this, this article reviews the biological origin of exosomes, related regulatory programs, their potential as biomarkers indicating *in vivo* drug exposure, and related research results in the above fields in recent years, aiming to deepen the understanding of the role of exosomes in drug metabolism and provide more reference for future research on individualized drug administration of exosomes.

## 1 Introduction

**FIGURE 1:**
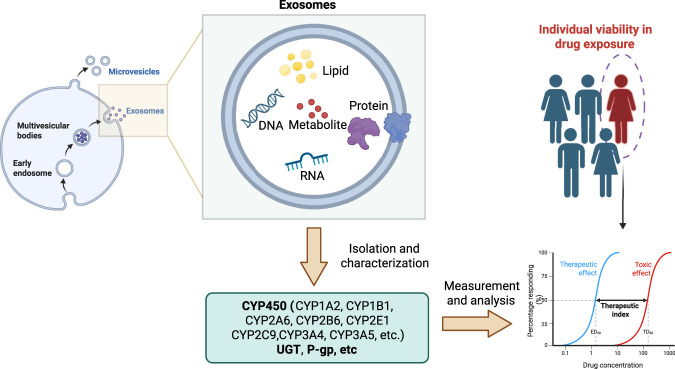
Exosomal drug metabolizing enzymes can be applied for monitoring *in vivo* drug exposure variability.

Cell-to-cell and cell-to-organ communication in biological systems is tightly regulated, and dysregulation of these processes can contribute to disease pathogenesis. Such communication can be mediated by exosomes—extracellular vesicles secreted by virtually all cell types and abundantly present in bodily fluids, including blood, urine, and saliva (Dimik et al., 2023). Exosomes carry diverse biomolecules, such as proteins, nucleic acids, lipids, and metabolic enzymes, which collectively reflect the physiological and pathological states of the organism (He et al., 2018; Jeppesen et al., 2019). As essential mediators of intercellular communication, exosomes play pivotal roles in numerous biological processes, including immunomodulation, tissue repair, and the maintenance of metabolic homeostasis (Xu et al., 2019). Pharmacogenomics presents as a fundamental tool for personalized drug therapy using genetic variation of drug-metabolizing enzymes to optimize drug therapy (Radosavljevic et al., 2023; Ingelman-Sundberg and Molden, 2025). Liquid biopsy-based monitoring of the gene expression of drug-metabolizing enzymes has been applied for therapeutic drug monitoring (TDM), and exosomes show great promise to serve as novel biomarkers in liquid biopsy (Yu et al., 2022). In recent years, increasing research revealed that exosome-derived protein and nucleic acid cargo can serve as potential biomarkers to define individual variability in drug exposure (Useckaite et al., 2021). In this review, we summarize the biogenesis, molecular cargo, and functional roles of exosomes, and discuss their emerging potential as novel biomarkers for variability in systemic drug exposure (Figure 1).

## 2 The biology and function of exosomes

### 2.1 Biological origin of exosomes

**FIGURE 2:**
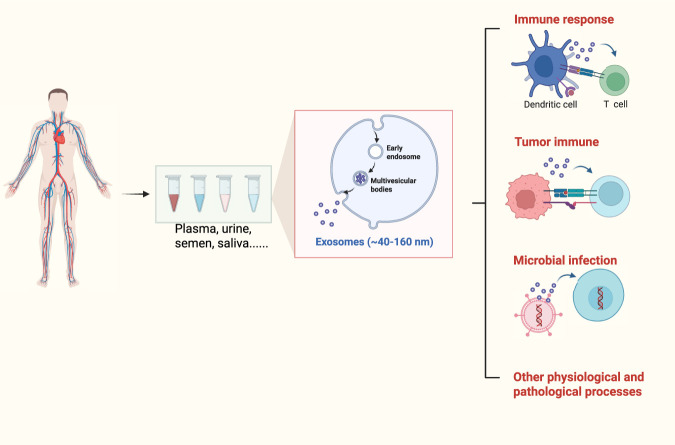
The origins and function of exosomes.

Exosomes are a subset of extracellular vehicles (EVs), typically 40–160 nm in diameter (with a mean around 100 nm), that originate from the limiting membrane of late endosomal compartments termed multivesicular bodies (MVBs) (Kalluri and LeBleu, 2020). Due to variations in cellular origin and physiological conditions, exosomes exhibit diverse compositions and biological functions. Exosomes are secreted by nearly all types of eukaryotic cells and found in various types of body fluids, including plasma, urine, semen, saliva, bronchial fluid, *etc.* (Doyle and Wang, 2019) (Figure 2). Massive research focused on the development of new technologies for exosomes separation, and the most commonly used include ultracentrifugation, size-based technologies, immunoaffinity-based capture, exosome precipitation, and microfluidics-based approaches (Doyle and Wang, 2019; Dilsiz, 2024; Shen et al., 2024; Welsh et al., 2024). Among them, differential ultracentrifugation was the first and most commonly used method for exosome isolation to date (Livshits et al., 2015). The sources of exosomes should also be considered before choosing optimal separation technologies.

Exosomes from various origins have also been widely applied in biomarker discovery and clinical monitoring. As they can be easily detected in most body fluids, exosomes are promising biomarkers in liquid biopsy (Yu et al., 2022). Thus, exosomes cannot only serve as targeted drug delivery carriers but also assist in early diagnosis of diseases, such as cancer (Moni et al., 2025). In normal physiological processes, exosomes act as a natural intercellular communication transporting cargo to regulate multiple cellular processes such as immune responses. Many types of immune cells can secrete exosomes, including dendritic cells, T cells, B cells, macrophages, and NK cells (Thery et al., 2009). Dendritic cell-derived exosomes carry MHC-peptide to activate specific T cells, and activated T cells can also release exosomes. For example, Treg cell-derived exosomes are reported to mainly participate in immunosuppression (Seo et al., 2018). Exosomes also play important roles in disease pathogenesis. Tumor cell-derived exosomes contribute to the immunosuppressive microenvironment to promote the progression of the tumor (Xie et al., 2022). While in microbial infection, exosomes exert complex roles as they can not only enhance immune responses by presenting pathogens but also facilitate infection by spreading pathogen-related molecules (Wang et al., 2018). Thus, exosomes can serve as an indicator to reflect the physiological or pathological state of the originating cells (Aheget et al., 2020).

The formation of exosomes initiates with the endocytic internalization of cargo and plasma membrane, progresses through multivesicular bodies (MVBs) maturation via selective cargo sorting and intraluminal vesicle (ILV) formation, and concludes with MVB-plasma membrane fusion and exosome release. Biogenesis of exosomes begins with clathrin-dependent or -independent endocytosis of surface receptors and membrane lipids, resulting in early endosome formation. The endosomal membrane then undergoes coordinated inward budding, facilitated by sequential recruitment of endosomal sorting complex required for transport (ESCRT) complexes, to generate ILVs. This membrane remodeling process culminates in the formation of MVBs—late endosomal organelles harboring numerous ILVs (Han et al., 2022). Cargo sorting into ILVs is mechanistically regulated by both ESCRT-dependent and ESCRT-independent pathways (Arya et al., 2024). The canonical ESCRT machinery comprises four distinct protein complexes, including ESCRT-0, -I, -II, and -III, that operate in conjunction with accessory proteins such as VPS4 ATPase, TSG101, and ALIX. ESCRT-0, primarily through hepatocyte growth factor-regulated tyrosine kinase substrate (HRS) and signal-transducing adaptor molecule (STAM), recognizes and sequesters ubiquitinated membrane proteins (e.g., activated receptor tyrosine kinases) within endosomal microdomains. This process serves as a signal for the sequential recruitment of ESCRT-I (via TSG101) and ESCRT-II (containing VPS25), which collectively promote membrane curvature and bud formation. ESCRT-III polymers then drive membrane constriction and facilitate the final abscission of ILVs into the endosomal lumen. Concurrently, the AAA + ATPase VPS4 disassembles ESCRT III and recycles the ESCRT machinery to ultimately produce ILV (Aheget et al., 2020; Gurung et al., 2021). ESCRT-independent mechanisms involve tetraspanins (e.g., CD63, CD81), lipids (such as ceramide), and chaperones like HSP90, which also contribute to membrane deformation and cargo selection during ILV biogenesis (Dai et al., 2020; Wiklander et al., 2019), initiating budding. ESCRT-III (VPS4/SnF7) drives membrane scission to form intraluminal vesicles (ILVs), and Alix protein assists nucleic acid loading. Furthermore, ESCRT-independent mechanisms also contribute significantly to intraluminal vesicle formation. These include the involvement of tetraspanin-enriched microdomains and ceramide-mediated membrane remodeling. For instance, neutral sphingomyelinase 2 (nSMase2) catalyzes the hydrolysis of sphingomyelin to generate ceramide, which promotes the inward budding of endosomal membranes specifically within tetraspanin (e.g., CD63/CD81)-enriched regions (Kalluri and LeBleu, 2020). Rab27a/b drives MVB transport to the plasma membrane, and Rab35 activates PLD2 to mediate membrane fusion. Finally, MVBs are transported to the plasma membrane via microtubules, dependent on the localization of the Rab GTPase family (Rab27a/b, Rab35) (Mahmoudi et al., 2023), and utilize the SNARE complex to mediate the fusion of multivesicular bodies (MVBs) with the plasma membrane, thereby secreting intraluminal vesicles (ILVs), or exosomes, into the extracellular space. This process is regulated by cellular metabolic status and stress-related signals. Thus, the formation of exosomes is precisely regulated by specific mechanisms and specific proteins, mRNA, and microRNA are packed into exosomes and delivered to different sites through biological fluids such as plasma (Krylova and Feng, 2023). For example, Kumar et al. first demonstrated the specific packaging of CYP enzymes in human plasma exosomes, highlighting the potential use of plasma exosomal CYPs as biomarkers.

### 2.2 Exosome cargo

Exosomes carry a diverse array of biomolecules, such as nucleic acids, proteins, lipids, and metabolites, with their specific composition being strongly influenced by the cell type and physiological state of the originating cells (Pegtel and Gould, 2019; Lai et al., 2022). In detail, nucleic acid components of exosomes include mRNA, miRNA, lncRNA, circRNA, as well as genomic and mitochondrial DNA fragments. For instance, exosomal miR-21 exerts complex roles in the pathogenesis of various diseases. Exosomal miR-21 can not only promote the proliferation, migration, and invasion of tumor cells, but also mediate chemoresistance and reshape the tumor microenvironment (Au Yeung et al., 2016; Cheng et al., 2022). Moreover, tubular cells derived exosomal miR-21 accelerates the development of renal fibrosis by targeting PTEN (Zhao S. et al., 2021). MiR-21 is also involved in the pathogenesis of ischemia-reperfusion injury and non-alcoholic fatty liver disease (NAFLD) (Xu et al., 2025; Hochreuter et al., 2022). Proteins constitute the largest proportion, accounting for approximately 60% of exosomal content, including universal exosomal marker proteins such as tetraspanins (e.g., CD63, CD81), which contribute to the structural stability of exosomes; ESCRT-related proteins such as Alix and Tsg101, which play key roles in regulating intraluminal vesicle (ILV) formation; heat shock proteins (e.g., HSP70, HSP90), which involved in protein folding and cellular stress responses, as well as tissue-specific proteins such as HER2 in breast cancer-derived exosomes and CD20 in lymphoma-derived exosomes, which can function as antibody “decoys” to neutralize therapeutic antibodies (Kalluri and LeBleu, 2020; Dai et al., 2020; Wiklander et al., 2019). Additionally, tumor cell-derived exosomal PD-L1 suppresses the activation of T cells to mediate immunosuppressive effects in anti-tumor immunity (Poggio et al., 2019). However, activated T cells that secreted exosomal PD-1 can attenuate PD-L1-mediated immunosuppression (Qiu et al., 2021). And neuronal exosomes contain neurofilament light and synaptophysin, both of which are neuronal markers (Sun et al., 2017). Meanwhile, the predominant lipid constituents of exosomes include cholesterol, sphingomyelin, and phosphatidylserine, which are involved in signal transduction and help maintain membrane integrity (Skotland et al., 2019). Moreover, exosome-derived metabolites and enzymes, such as lactic acid and short-chain fatty acids, can acidify the local microenvironment and induce immunosuppression, and exosomes associated with neurodegenerative diseases carry enzymes involved in amyloid-β oligomer processing, such as endothelin-converting enzyme 1 and 2 (ECE1/2) (Meng et al., 2020). Furthermore, engineered exosomes can be loaded with exogenous therapeutic cargo, including siRNA, chemotherapeutic agents (e.g., paclitaxel), and the CRISPR/Cas9 system for targeted delivery due to high biocompatibility, low toxicity, and low immunogenicity (Kar et al., 2023).

### 2.3 Functions of exosomes

Exosomes serve as crucial mediators of intercellular communication. They are internalized by recipient cells via mechanisms such as surface ligand-receptor binding, membrane fusion, or endocytosis, thereby delivering their bioactive cargo and modulating physiological processes in the target cells (Singh et al., 2024).

In the context of immunomodulation and host defense, dendritic cell (DC)-derived exosomes carry major histocompatibility complex class II (MHC-II) and co-stimulatory molecules (e.g., CD86), which contribute to the activation of T cell-mediated immune responses (Yu et al., 2021). Exosomes secreted by regulatory T cells suppress CD8^+^ T cell proliferation, thereby attenuating immune activation, and deliver anti-inflammatory factors such as TGF-β and IL-10 to inhibit macrophage-mediated inflammatory responses (Zhang et al., 2020). Mesenchymal stem cell (MSC)-derived exosomes carry specific miRNAs—such as miR-133, to promote myoblast differentiation and muscle regeneration (Yue et al., 2020). Moreover, MSC-derived exosomal TSG-6can inhibit NF-κB signaling and mitigate myocardial ischemia-reperfusion injury, thereby facilitating cardiac tissue repair (Mahmoudi et al., 2023). Besides, neuronal exosomes contain miR-132, which upregulates synaptic protein expression via the CREB pathway. Microglia transfer miR-146a-5p via exosomes to inhibit neurogenesis within the hippocampal dentate gyrus (DG) by directly targeting KLF4 (Fan et al., 2022). In terms of microbial interactions, macrophage-derived exosomes deliver miR-155 into infected cells to restrain TLR4 pathway-mediated hyperinflammation (Macia et al., 2019). Epithelial exosomes encapsulate antimicrobial peptides (e.g., defensins) capable of direct pathogen killing (Meng et al., 2020) and hepatitis viruses exploit the exosomal ALIX pathway to package viral RNA into vesicles and evade immune clearance (Wiklander et al., 2019). *Mycobacterium tuberculosis* upregulates host exosomal miR-21 to enhance autophagy escape and improve bacterial survival (Macia et al., 2019). Adipocyte-derived exosomes deliver miR-27a to inhibit PPARγ and modulate hepatic insulin signaling. Moreover, studies in obese mice indicate that exosomal miRNAs such as miR-122, miR-192, and miR-27a-3p can suppress PPARα and accelerate insulin resistance and dyslipidemia (Castano et al., 2018; Tang et al., 2023). All in all, exosomes exert complex but significant roles in a plethora of physiological and pathological processes.

## 3 The potential of exosome characterization in biomarking drug exposure variants

As key mediators of intercellular communication, exosomes have contributed significantly to discoveries in basic research and are increasingly being applied in disease diagnosis and treatment (Zhang et al., 2020). For example, cancer cell-originated exosomes can be detected by Surface-Enhanced Raman spectroscopy (SERS) for cancer early diagnosis (Liu Y. et al., 2024). Although research on exosome-based drug delivery systems and exosomes as disease biomarkers continues to advance, studies focusing on exosome-derived metabolic enzymes remain limited. In recent years, metabolic enzyme markers from exosomes have the potential to directly correlate enzyme activity with disease phenotypes, offering considerable diagnostic value in clinical applications, particularly in exosome-related cancers, metabolic disorders, and neurodegenerative diseases. This novel perspective promises to yield new insights into the unique roles of exosomes in metabolic processes and enhance our understanding of metabolic heterogeneity in diseases.

Exosomes are important mediators of intercellular communication, and the diversity of their origin directly determines their applications in drug exposure variability monitoring. A large number of studies have shown that plasma exosomes are mainly derived from various types of tissue cells and exert significant functions in a plethora of diseases such as cardiovascular diseases (Bellin et al., 2019), liver diseases (Luo et al., 2022), metabolic diseases (Sun et al., 2021), and cancer (Casagrande et al., 2023). The dynamic crosstalk between exosomes originating from different tissues is also essential in maintaining physiological homeostasis or accelerating diseases progression. Cardiovascular-derived exosomes secreted by cardiac progenitor cells can protect musculoskeletal tissues against inflammation and muscular dystrophy, while musculoskeletal-derived exosomes can also promote cardiac repair and regeneration (Li et al., 2025). Cancer cell-secreted exosomes can also affect neighboring non-tumor recipient cells to influence tumor progression (Becker et al., 2016). Bioactive molecules encapsuled by exosomes, such as mother cell-specific metabolic enzymes, transport proteins, and nucleic acids, can not only redirect the recipient cells, but also enable exosomes as potential biomarkers to monitor physiological processes or disease status (Mallia et al., 2020). Exosomes can directly modulate the profile of medication PK/PD, and exosomes containing drug-metabolizing enzyme can serve as unique molecular fingerprints of drug metabolism variants among different individuals (Kalluri and LeBleu, 2020; van Niel et al., 2018; Liu J. et al., 2024).

**FIGURE 3:**
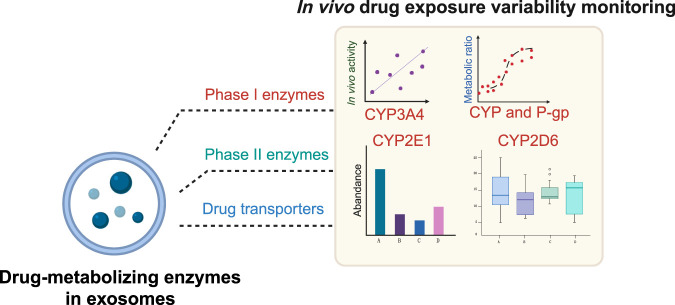
Drug-metabolizing enzymes in exosome can be used as biomarkers to monitor drug exposure variants.

Currently, the differences in the expression and activity of drug-metabolizing enzymes are considered to primarily determine the variation of drug exposure *in vivo* (Figure 3) (Almazroo et al., 2017). Phase I metabolic enzymes are responsible for converting lipophilic chemicals to hydrophilic products. Cytochrome P450s (CYP450s), as the most representative Phase I enzymes, are involved in the metabolism of more than 70% of drugs (Zhao M. et al., 2021). High variation in the expression or redox reaction activity of phase I metabolic enzymes such as the CYP450 family can lead to a difference of dozens of times in drug clearance (Hossam et al., 2024). Although hepatocytes are the main sources of CYP450s, the expression of CYP450s has been widely discovered in various extrahepatic tissues, including the small intestine, kidney, lung, heart, etc. Moreover, CYP450s can be packed into exosomes and secreted from liver and other peripheral organs, and delivered to distant sites to affect extrahepatic drug metabolism, detoxification (Gerth et al., 2019) **.** Changes in drug gene polymorphism can affect conjugation reactions mediated by phase II conjugating enzymes, such as UGT and GST to influence the clearance of drugs (Kane et al., 2023; Aloke et al., 2024). The transport of reactive metabolites across the cell membrane is mediated by drug transporters such as P-gp and OATP, and their dysregulation will disturb the absorption and distribution of drugs and play important roles in drug resistance the adverse effects (Guo et al., 2024; Balasubramanian and Maideen, 2021). Thus, it is evident that those three major categories of metabolic enzymes as key nodes in drug ADME are affected by a variety of factors, and the variation in their expression and function leads to drug exposure variability, and are also indicative markers for guiding drug ADME variation.

Based on the above description, although HLA genotyping or metabolite markers detection are currently the main approaches for explaining drug exposure variability, there are still some defects in them. HLA genotyping can identify some genetic polymorphisms, such as CYP2D6, UGT1A1*28, *etc.*, but the explanation for phenotypic variation is only 30%–50%, and it cannot predict dynamic changes caused by environmental factors such as drug interactions and disease states. Tissue biopsy can directly quantify the amount of enzyme protein, but its invasiveness limits repeated sampling, and local sampling cannot accurately reflect the overall state of the organ. The endogenous metabolites of P450 can also serve as potential biomarkers and 6β-hydroxycortisol and 4β-hydroxycholesterol are the most well-known among them. But the response of some metabolite markers in plasma, such as 4β-hydroxycholesterol, is relatively slow and cannot provide real-time indication of disease changes. All of the above methods cannot well reflect the spatiotemporal dynamics of drug exposure variation and the multi-organ interaction network (Achour et al., 2022; Drozdzik et al., 2021; Perepechaeva et al., 2024; Lee et al., 2019). In contrast, exosomes can dynamically monitor drug exposure from the local tissue to the overall level. In particular, exosomes can integrate gene expression, post-translational protein modifications, and environmental regulation information to reflect tissue-specific metabolic states, and correct drug metabolism affected by both genetic and non-genetic factors, providing a multi-dimensional biomarker system for constructing individualized medication models (Rowland et al., 2019). Thus, using exosomes to reflect the ADME of drugs has been proposed as a non-invasive liquid biopsy to characterize interindividual variability of drugs (Table 1).

**TABLE 1:** Representative drug-metabolizing enzymes in exosome serve as biomarkers to monitor drug exposure variants.

Metabolic enzymes in exosomes	Related drug exposure variants	Clinical significance	References
CYP3A4	Strong concordance between exosome-derived CYP3A4 protein and midazolam CL/F	Demonstrating that human plasma-derived exosomes can serve as liquid biopsy	Kane et al. (2023)
CYP2E1	Significantly induced by alcohol in liver injury	As a potential biomarker for alcoholic liver injury	Aloke et al. (2024); Balasubramanian and Maideen (2021)
CYP3A4	Pregnancy-related hormones (PRH) induce CYP3A4 expression to increase nifedipine metabolism	Hepatocyte-derived exosomes might serve as biomarkers of hepatic CYP3A4 metabolism	Rowland et al. (2019)

As early as 2017, Kumar et al. first isolated exosomes from plasma and analyzed the expression and activity of CYP enzymes in plasma exosomes. CYP1B1, CYP2A6, CYP2E1, and CYP3A4 were detectable in plasma exosomes and the mRNA of CYP2E1 was more than 500-fold higher than that of other CYPs (Kumar et al., 2017). Subsequently, Achour et al. performed plasma exosomal transcriptome analysis and discovered that the mRNA levels of 12 key drug-metabolizing enzymes and four drug transporters are highly positively correlated with their protein levels in the liver, which can be used to stratify patients for drug dose adjustment (Achour et al., 2021). Furthermore, Rowland et al. successfully isolated exosomes from normal human plasma of six healthy Caucasian males aged 21–35 years via ultracentrifugation. And the presence of CYP enzymes, UGT and NADPH-cytochrome P450 reductase was further demonstrated in plasma exosomes. Moreover, their results demonstrated a statistically significant positive correlation between exosomal CYP3A4 mRNA (R^2^ = 0.7874) and protein (R^2^ = 0.9045) levels and oral midazolam clearance, providing compelling evidence that exosomes carry functionally relevant CYP450 transcripts and proteins, which present as a viable alternative to tissue biopsy (Rowland et al., 2019). The above studies established, for the first time, the potential of exosomal biomarkers as non-invasive substitutes for liver biopsy in the quantification of metabolic enzyme activity, thereby inaugurating a novel approach for personalized pharmacotherapy and stimulating subsequent investigations under pathological conditions. In 2017, Cho et al. reported that the number of exosomes and the abundance of exosomal CYP2E1, CYP2A, CYP1A1/2, and CYP4B proteins were significantly elevated in both alcoholic patients and alcohol-exposed rodents. Alcohol inducible CYP2E1 elevated exosomes and exosomal P450 enzymes to mediate oxidative hepatocyte injury, which revealed an alternative role for elevated exosomal CYP2E1 as a potential biomarker for liver injury (Cho et al., 2017). Moreover, Cho et al. also discovered that exosomes isolated from mice with acetaminophen (APA)-induced liver injury can also cause damage to the recipient hepatocytes and mice (Cho et al., 2018). Similar results were consolidated by Kumar et al. They also found that plasma exosomal CYP2E1 mediated alcoholic or APA-induced toxicity (Kumar et al., 2017). Kumar et al. employed enzyme-linked immunosorbent assay (ELISA) to quantify CYP2E1 levels in plasma exosomes derived from 40 patients diagnosed with alcoholic liver disease, with parallel measurements obtained from liver tissue biopsies. Their analysis revealed a 3.2-fold elevation in exosomal CYP2E1 content within the alcohol-exposed cohort, which exhibited a positive correlation with histopathological liver injury scores (r = 0.65). Notably, the increase in exosomal CYP2E1 was preceded by elevated ALT levels by several months (*P* < 0.001), supporting the proposition that exosomal CYP2E1 may serve as an early predictive biomarker for incipient alcoholic hepatotoxicity. These studies emphasized that the role and application of exosomal CYP2E1 as potential biomarkers for alcohol- and drug-induced liver injury. Moreover, Brahim Achour et al. verified that the exosomal expression of CYP1A2, CYP2B6, CYP2C9, CYP3A, and P-gp correlated with their activity phenotype (r = 0.44–0.7, P<0.05). This study confirmed that the detection of the expression of exosomal CYP enzymes and P-gp based on liquid biopsy shows great promise to realize quantitative measurement of individual drug elimination capacity (Achour et al., 2022) **.** The following year, Cho et al. conducted *in vitro* metabolic assays by incubating plasma exosomes from lung cancer patients with erlotinib, followed by metabolite profiling using UPLC. Their results indicated a 40% increase in drug clearance rates in groups exhibiting high exosomal CYP1A1 expression (*P* < 0.01), suggesting that tumor cells actively secrete functional CYP enzymes via exosomes (Zhang et al., 2020; Alfieri et al., 2011). These observations imply a hitherto unrecognized mechanism of exosome-mediated targeted drug resistance, wherein exosomes may play an active role in drug metabolism prior to their release rather than merely serving as vehicles for passive transfer, which contrasts with earlier reports by Kumar et al., In 2024, Achour et al. utilized mass spectrometry to simultaneously quantify the protein expression of eight CYP enzymes within exosomes isolated from 30 patients with cardiovascular disease. Their analysis revealed a significant correlation between exosomal CYP1A2, 2B6, 2C9, 3A4, and 3A5 protein levels and measurements of activity, and a multi-enzyme synergistic model accounted for 45% of interindividual variability in drug dosage (Achour et al., 2022). This work represents a conceptual shift from single-enzyme analyses toward integrated proteomic profiling, thereby contributing a multidimensional dataset essential for constructing individualized metabolic phenotypes. Rodrigues et al. also demonstrated the dynamic monitoring of CYP induction by tracking temporal changes in exosomal CYP3A4 mRNA following rifampicin administration. They observed a 3.7-fold induction of CYP3A4, the kinetics of which closely paralleled alterations in midazolam AUC (*r* = 0.85) (Rowland et al., 2019). Moreover, Khatri et al. revealed that the mRNA levels of hepatocyte exosomal CYP3A4 were positively correlated with CYP3A4 protein levels and nifedipine metabolism and might serve as biomarkers of nifedipine metabolism and clearance in gravida (Khatri et al., 2021). Notably, exosomal mRNA responses preceded changes in systemic drug concentrations, affirming the utility of exosomal biomarkers for real-time assessment of enzyme induction. This dynamic profiling paradigm complements the static abundance measurements reported by Achour et al., collectively constituting a comprehensive framework for metabolic evaluation that integrates both basal expression and adaptive pharmacological responses.

Apart from CYP enzymes, other drug-metabolizing enzymes, including Phase II enzymes and drug transporters present in exosomes, also play crucial roles in *vivo* drug variability. Rowland et al. demonstrated the presence of UDP-glucuronosyltransferase (UGT) proteins and mRNAs in isolated human plasma exosomes, specifically highlighting their role as biomarkers in characterizing variability in drug exposure (Rowland et al., 2019). Moreover, exosomes carrying drug metabolizing enzymes or transporters also participate in mediating chemoresistance. Exosomes can transfer P-gp, a well-known protein in cancer drug resistance, to the recipient cancer cells and lead to acquired drug resistance (Guo et al., 2024; Rowland et al., 2019; Tang et al., 2021; Li et al., 2020). It has been reported that GSTO1-1 could be released by cancer cells through the exosomal route to affect the cisplatin resistance in different types of cancer cells, highlighting the presence and function of exosomal UST in drug resistance (Piaggi et al., 2024). Rigalli et al. constructed a UPLC-MS/MS method to validate EVs as surrogates to regulate the activity of drug transporters in tumor cells, focusing on ABCC2 (MRP2) and ABCG2 (BCRP) (Rigalli et al., 2024). Multiple studies also have shown that exosomes can transfer multidrug resistance (MDR) phenotype between tumor cells via the delivery of ABCB1 (Torreggiani et al., 2016; Wang et al., 2016). Drug metabolism even can occur within the exosomes prior to their release into plasma or uptake of exosomes by target cells. Anti-tumor compounds can be sequestered into exosomes or accumulated in the membrane of exosomes, thus contributing to drug resistance (Conde-Vancells et al., 2010).

Over the past decade, research in this field has achieved transformative advances, marked by three major leaps: from single-enzyme detection to multi-enzyme network analysis, from static abundance measurements to dynamic activity monitoring, and finally, from laboratory-based assays to point-of-care rapid testing. Exosome-based technologies are rapidly advancing toward high-precision applications, with growing clinical evidence supporting their value (Enlo-Scott et al., 2021). These developments position exosome analysis as a cornerstone technology for the next-generation of personalized therapeutics.

## 4 Discussion

Exosomes are a class of nano-sized vesicles that can be produced and secreted extracellularly by the vast majority of cells (Singh et al., 2024). They are messengers for communication between cells. They selectively package bioactive substances such as proteins, nucleic acids, and lipids obtained from the parent cell, and transport these substances into the extracellular circulatory system for exchange and transmission, thereby achieving the function of regulating many physiological and pathological processes (Han et al., 2022). This natural carrying capacity enables it to serve as a biomarker describing the real-time status and internal changes of tissue and cell microenvironment in complex biological environments such as drug exposure, providing a new opportunity for non-invasive, dynamic, and precise monitoring of drug behavior *in vivo*. Currently, the most commonly used methods in clinical practice still have shortcomings such as strong invasiveness and difficulty in capturing complete tissue-specific dynamics, especially when tissue differences are significant. Therefore, there is an urgent need to establish a series of more stable, less invasive biomarkers with tissue/cell-specific recognition functions that can reflect the overall environment of drug action.

Current studies have shown that exosomes contain a plethora of drug-metabolizing enzymes, which can reflect the information of the parent cell that changes over time. This not only overcomes the disadvantages of traditional blood drug concentration detection methods, such as high instantaneousness and frequent sampling, but also provides a revolutionary possibility for non-invasive monitoring of drug exposure in tissues. Therefore, the value of plasma exosomes as a novel, dynamic, and tissue-specific marker reflecting inter-individual differences in drug exposure needs to be further explored. Using exosome analysis technology to solve the problem of inter-individual pharmacokinetic variability will be a new breakthrough point in the future. Thus, it cannot be overlooked to choose the optimal analytical methods to detect and quantify exosomal drug-metabolizing enzymes. LC-MS/MS-based proteomic screen for exosomes can reveal the full view of drug metabolizing enzymes in exosomes. And ELISA, qPCR and Western blot can be used for the quantification of the expression specific exosomal enzymes. In recent years, a plethora of microfluidic platforms have been developed for the isolation and detection of exosomes, which may further benefit the precision analysis of exosomal drug metabolizing enzymes (Doyle and Wang, 2019; Raju et al., 2022). At the same time, the effect of pre-analytical variables on the yield and integrity of exosomal drug metabolizing enzymes should also be carefully considered. It has been reported that blood collection tubes, incubation or transportation time before the initial centrifugation, centrifugation method, and storage temperature have great impacts on the phenotype of exosomes in blood (Bæk et al., 2016; Batool et al., 2023).

With dynamic changes in body fluids, the characteristics and contents of exosomes are highly associated with physiological and pathological states such as age, race, and sex (Noren et al., 2022). Since drug exposure variability is also affected by a series of complex factors, it cannot be overlooked to consider the influence of physiological and pathological states, drug-drug interactions, genetic polymorphisms, and even gut microbiome when using exosomal metabolic enzymes to monitor drug exposure variability. Moreover, the changes in the expression of exosomal metabolic enzymes can also serve as biomarkers for disease states and reflect the variability of drug-drug interactions in non-invasive manners. Thus, it is valuable to integrate the change of exosomal metabolic enzyme and other pharmacokinetic factors to comprehensively explore drug exposure variability *in vivo*. Moreover, it remains unclear that the exact role of exosomal phase II metabolic enzymes and drug transporters in drug metabolism variability. Thus, more effort should be focused on the characterization of exosomal enzymes and the evaluation of their effect on *in vivo* drug metabolism variability.

Since pharmacogenomics technologies provide information on genetic predisposition, they can not necessarily reflect real-time drug metabolism activity. On the other hand, plasma exosomal enzyme biomarkers can reflect the real-time drug variability as a non-invasive liquid biopsy, but often lack tissue-specific information. Thus, in the future, it is also necessary to combine exosome technology with pharmacokinetic models, artificial intelligence prediction models, and other methods to achieve comprehensive and accurate evaluation, and further realize the evaluation method that has risen from the previous single drug concentration evaluation method to the current comprehensive judgment of drug action, so as to provide a reliable basis for the clinical practice of the “new era of individualized precision medication” in the future and provide patients with better treatment options.
